# Serotransferrin enhances transferrin receptor-mediated brain uptake of antibodies

**DOI:** 10.1007/s13346-025-01811-1

**Published:** 2025-02-19

**Authors:** Jamie I. Morrison, Nicole G. Metzendorf, Jielu Liu, Greta Hultqvist

**Affiliations:** Institutionen För Farmaci, Uppsala Universitet, Uppsala, Sweden

**Keywords:** Antibody, Blood−brain barrier, In vitro BBB, In vivo BBB, Serotransferrin, Transcytosis, Transferrin receptor

## Abstract

The propensity of antibody-based therapies to systemically enter the brain interstitium and ameliorate pathology associated with numerous neurological maladies is precluded by the presence of the blood–brain barrier (BBB). Through distinct mechanisms, the BBB has evolved to regulate transport of essential ions, minerals, certain peptides and cells between the blood and the brain, but very restrictive otherwise. Hijacking receptor-mediated transport pathways of the BBB has proved fruitful in developing “Trojan Horse” therapeutic approaches to deliver antibody-based therapies to the brain milieu. The transferrin receptor (TfR)-mediated transcytosis pathway (RMT) is one such example where large recombinant molecules have been designed to bind to the TfR, which in turn activates the RMT pathway, resulting in delivery across the BBB into the brain milieu. Based on these findings, we here investigated whether the addition of serotransferrin could trigger the endogenous TfR-mediated RMT pathway and hence be used to enhance the uptake of TfR binding antibodies. By using an in vitro model of a mouse BBB we could test whether co-administration of mouse serotransferrin with mouse and human-based monoclonal antibodies enhanced brain uptake. In all cases tested, no matter if the monoclonal antibodies were designed to bind the TfR in a monovalent, partially monovalent/bivalent or entirely bivalent fashion, with high or low affinity or avidity, the addition of mouse serotransferrin significantly improved transport across the artificial BBB. This was also true for TfR binding antibodies that on their own passes the BBB poorly. These results were subsequently confirmed using a human in vitro BBB model, along with human serotransferrin and human TfR-binding antibody. To corroborate the in vitro results further, we conducted two pilot in vivo brain uptake study in wildtype mice, by intravenously co-administering a monoclonal TfR-binding antibody in the presence or absence of mouse serotransferrin as a proof-of-concept. In a similar outcome to the in vitro studies, we observed a significant almost two-fold increase in uptake of two different TfR binding antibodies in the brain when it was co-administered with mouse serotransferrin. These findings show for the first time that serotransferrin supplementation can significantly improve the ability of TfR-binding antibodies to traverse the BBB, which provides a realistic therapeutic opportunity for improving the delivery of therapeutic antibodies to the brain.

**Graphical Abstract:**

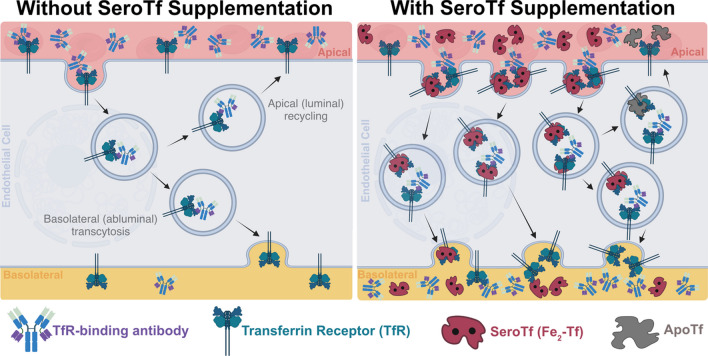

**Supplementary Information:**

The online version contains supplementary material available at 10.1007/s13346-025-01811-1.

## Introduction

Maintenance of cellular iron homeostasis is essential for a multitude of different processes around the body, requiring tight regulation of the transport of this metal ion into and out of the cell at physiologically pertinent periods [2]. One such organ where iron homeostasis is important is the brain. In addition to key holistic functions such as oxygen transport and DNA synthesis, iron is an essential cofactor in critical neurological functions such as, myelination, neurotransmitter synthesis and energy production [2]. Unlike many other organs and tissues within the body, transport of iron to the brain is hindered by the blood brain barrier (BBB), a selectively permeable endothelial cell layer whose main role is to ensure regulated nutrient entry, while maintaining a barrier against unwanted entities such as pathogens [55]. Even though neuropathological conditions as a result of disruptions to iron homeostasis in the brain are unequivocal [15], the process behind maintaining neurological iron levels is poorly understood.

Transferrin is an essential protein for regulating the absorption, utilization, recycling and storage of heme–iron. Once dietary iron enters the bloodstream, transferrin binds it, enabling shuttling of the metal systemically to all tissues around the body. Apo-transferrin (lacking iron) can bind two atoms of cellular impermanent ferric iron (Fe^3+^), forming a di-ferric-transferrin complex (Fe_2_-Tf – serotransferrin). Serotransferrin is delivered to cells through binding to the transferrin receptor 1 (TfR) [30, 41]. Once bound to the TfR, the Fe_2_-Tf complex undergoes endocytosis, whereupon a H^+^-ATPase-mediated acidification of the endosome leads to Fe_2_-Tf complex destabilisation [24, 30, 34]. As a result of the destabilization, Fe^3+^ is released from the transferrin, reduced to the more metabolically available ferrous iron Fe^2+^ and released into the cytoplasm where iron-chaperone proteins are employed to assimilate and/or store the iron [21, 38]. The apo-transferrin and TfR is recycled out of the cell and back to the cell surface respectively, making themselves available to begin the whole process anew.

Even though the aforementioned process is synonymous with iron homeostasis in erythrocytes, similar pathways are active when storing, assimilating and transporting iron through the endothelial cells that make up the BBB [1]. The process of guiding iron through endothelial cells layer of the BBB into the brain, rather than storing or assimilating the iron, is referred to as TfR iron-mediated transcytosis. The main pathway likely begins with serotransferrin binding to the TfR residing on the luminal surface of the BBB endothelial cells. As has been shown for canonical cellular iron uptake, the serotransferrin undergoes endocytosis into the cell. However, instead of going through the routine of ferric iron reduction and apo-transferrin recycling to the bloodstream, the Fe_2_-Tf complex enigmatically traverses the cytoplasm and is transcytosed, resulting in the release of iron into the abluminal brain milieu. While the precise mechanism behind TfR-RMT remains to be elucidated, it has been shown previously that the endothelial cells of the BBB are major players in how brain iron homeostasis is regulated [5, 49].

The entire process of binding TfR and triggering RMT has provoked scientists around the world to question whether the TfR-mediated RMT pathway could be used as a possible route for delivering biologics into the brain. Some of the first proof-of-concept published studies to address this possibility were published in 1987, where transferrin peptides were shown to traverse the BBB using RMT pathways [16]. This catalysed further studies demonstrating the possibility of non-invasively delivering large macromolecular TfR targeting antibodies to the brain via RMT pathways [37]. Fast forward 30 years and the TfR-mediated RMT pathway is one of the most attractive options for delivering biopharmaceuticals to the brain [51]. Due to the prominent expression of TfR on the luminal endothelial cell surface [36] and the identification of TfR binding proteins such as 8D3 [3, 25], the TfR-RMT pathway has been successfully targeted to act as a conduit for delivering large protein payloads, such as antibodies, across the BBB [4, 7, 20, 42, 43]. “Trojan Horse” tactics target the TfR, resulting in the successful delivery of large, macromolecular biopharmaceuticals across the BBB via TfR-RMT pathways. In light of this recent evidence, a fascinating interplay begins to take shape between the canonical Fe_2_-Tf BBB transport pathways and the transport of therapeutical proteins targeting the TfR. If we can agonistically provoke canonical Fe_2_-Tf uptake into the endothelial cells of the BBB, could this possibly improve RMT of TfR targeting protein-based therapeutics?

In order to investigate this, we utilised a modified In-Cell BBB-Trans assay, which is an in vivo validated artificial murine in vitro BBB model system that allows the user to effectively assess the transcytosis efficacy of TfR-binding antibodies (Morrison, Petrovic, et al., 2023). Here we demonstrate that supplementing cEND with mouse serotransferrin, previously pulsed with TfR-binding-8D3 antibodies, resulted in a significant enhancement of transcytosis of the antibodies. Furthermore, supplementing cEND cells with mouse serotransferrin also significantly enhanced transcytosis of TfR binding antibodies that transport negligibly through the BBB. In addition, using a modified human In-Cell BBB-Trans assay, replacing cEND cells with human endothelial cells (hCMEC/D3), we were able to show a significant enhancement of transcytosis for an antibody binding to human TfR when supplementing with human serotransferrin. An in vivo brain uptake proof-of-concept study could further confirm the results.

In conclusion, we demonstrate the additive effects of supplementing both mouse and human TfR-binding BBB-penetrating antibodies with species specific serotransferrin results in greater transcytosis efficacy and subsequent brain uptake. This finding holds great promise in improving the brain uptake of therapeutic antibodies that utilise the TfR-RMT pathway, significantly enabling most TfR binders to work as a BBB transporter and enhancing the efficacy of TfR binders that are already functional as BBB transporters.

## Results

### Purified mouse serotransferrin binds mouse and human TfR

**Fig. 1:**
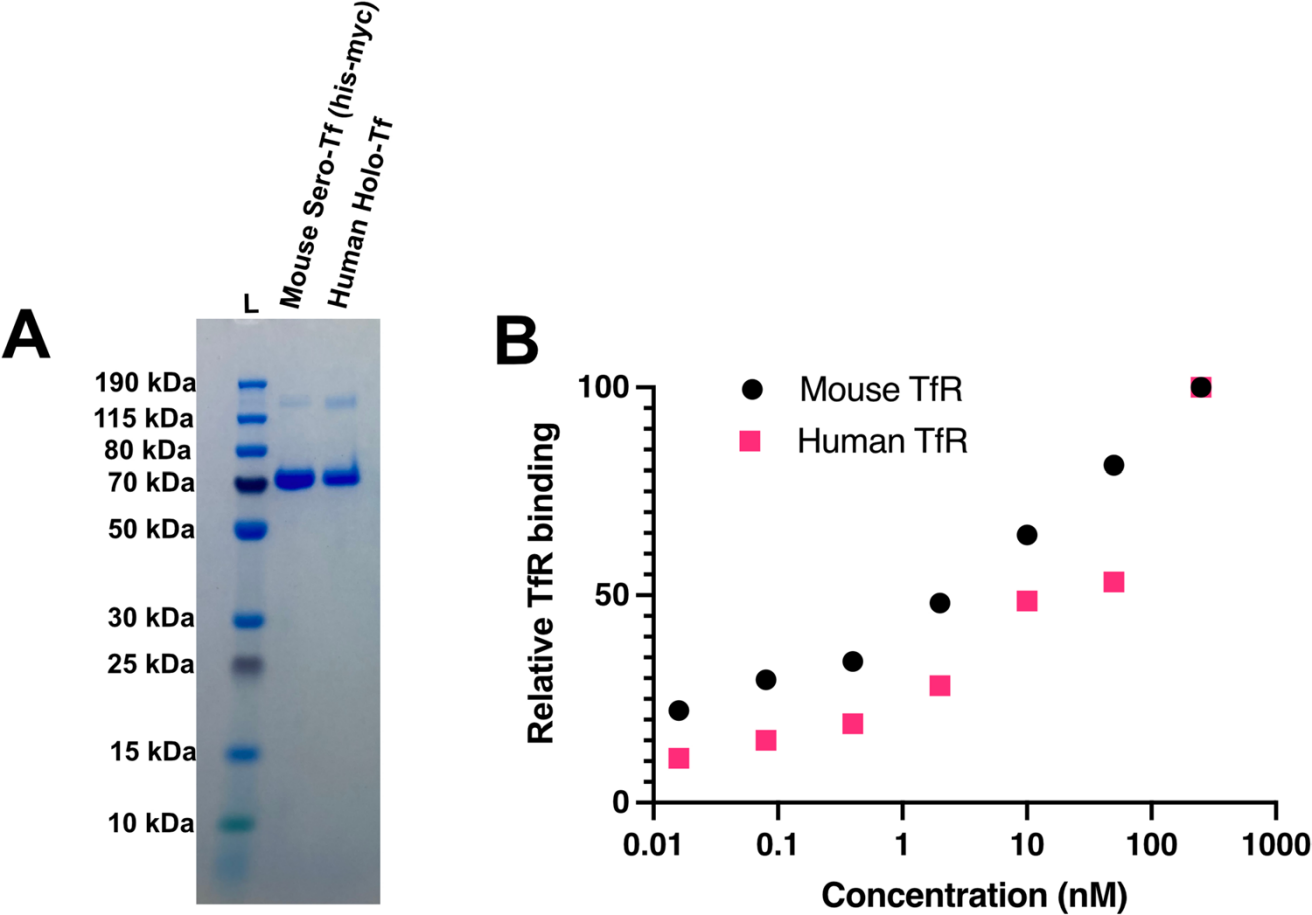
A. SDS-PAGE gel analysis of the purified recombinant mouse (Mouse Sero-Tf (his-myc)) and Human Holo-Tf in non-reducing conditions. A pre-stained ladder (L) was used to determine the approximate molecular weight corresponding to mouse serotransferrin. **B**. ELISA data representing the binding efficacy of mouse serotransferrin to both the mouse and human TfRs. NOTE: the 1000 nM data point for the human TfR is masked by the 1000 nM data point for the mouse TfR

In order to test whether the efficacy of supplementing with mouse serotransferrin improves brain uptake of recombinant monoclonal antibodies binding to TfR, we designed the mouse serotransferrin based upon a 697 amino acid sequence deposited in Ensembl (ENSMUST00000112645.8). The sequence was modified to include a N-terminal his and myc tag (his-myc mouse serotransferrin), in order to allow for downstream purification and in vitro detection of the protein. Following transient transfection in EXPI293 cells, the modified serotransferrin was successfully purified using a nickel column. An SDS-PAGE analysis of the purified protein, along with human serotransferrin (human holo-transferrin), revealed a single band at approximately 77 kDa (Fig. 1A), which corresponds to the molecular weight of the mouse serotransferrin protein and human serotransferrin. To ensure the tag modifications did not interfere with the TfR interaction of the recombinant mouse serotransferrin, a qualitative mouse TfR ELISA was performed, clearly showing binding (Fig. 1B). Interestingly, the mouse serotransferrin also demonstrated an ability to bind to human TfR, albeit with a lower binding efficiency when compared to mouse TfR.

### Antibodies to test

**Fig. 2:**
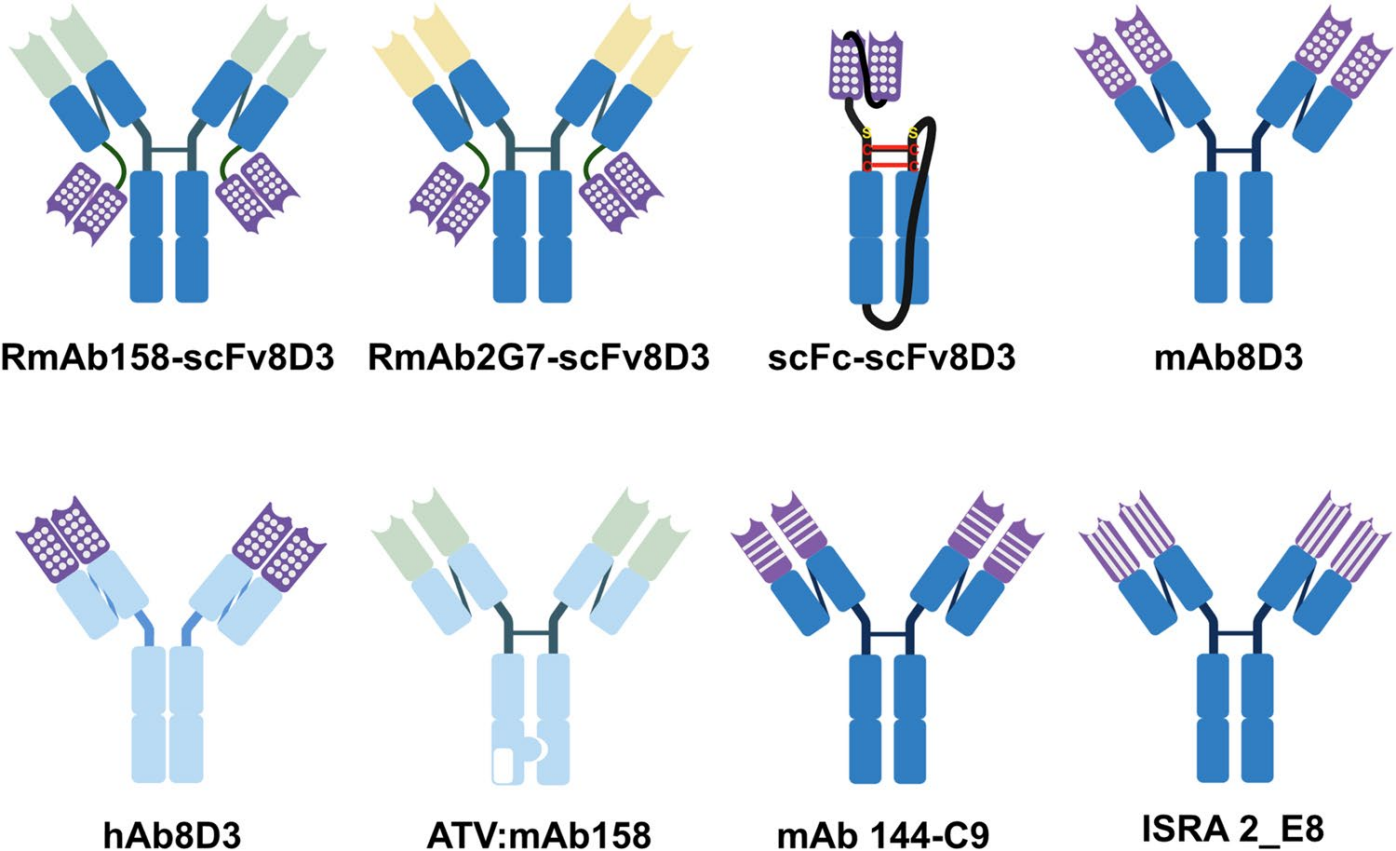
A schematic overview of the antibodies used in this paper. The light blue constant part represents a human IgG backbone while the dark blue is murine. Purple variable regions bind to TfR. The doted ones come from the on the 8D3 antibody. The striped ones are ones developed by us or our collaboration partners. The ATV binds TfR with a part of the Fc

In order to test if serotransferrin affects the transcytosis across the BBB of TfR binding antibodies, a repertoire of antibodies is needed. A schematic overview of the design of the antibodies we used is presented in Fig. 2. All antibodies were produced in EXPI293 cells. An SDS page of the purity of the antibodies can be found in supplementary Fig. 2.

### Removing the inhibitory effect of bovine serum improves basolateral transcytosis

**Fig. 3:**
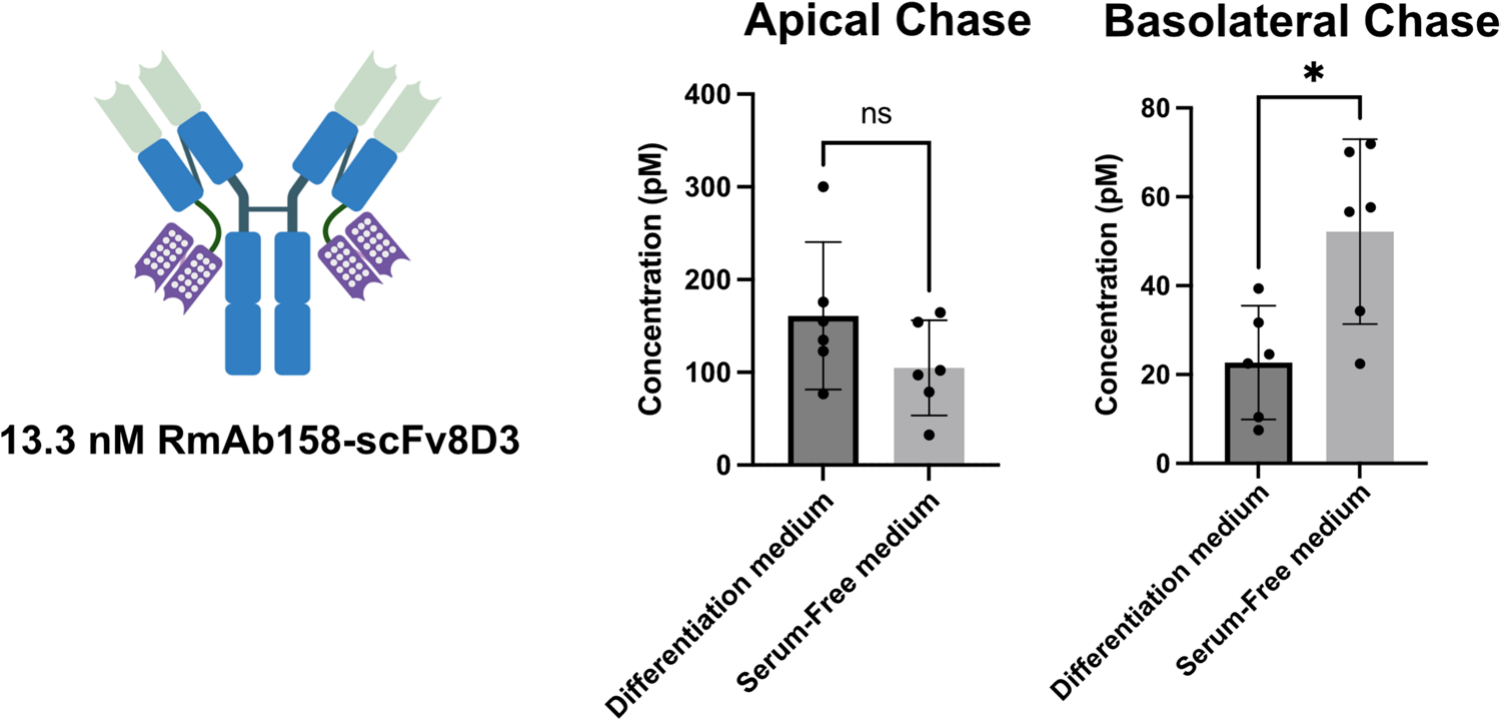
Cartoon representations of the RmAb158-scFv8D3, along with the graphical representation of average antibody concentrations found in the apical and basolateral 6-h chase compartments of cEND cells (Passage 13) plated on 0.4 µm translucent pore Bio-One® 24-well transwell cultures, primed for three-days in either differentiation or serum-free medium and followed by a one-hour “pulse” with 13.3 nM RmAb158-scFv8D3. Six transwells were used for each pulsed antibody condition (n = 6 technical replicates). The error bars represent 95% confidence intervals. Non-parametric Mann–Whitney pairwise comparisons were conducted as indicated. * Represents a significance level of P < 0.05. The polka-dotted regions on the cartoon represent the recombinantly added scFv8D3 that binds to the TfR

The validated in vitro murine BBB model system In-Cell BBB-Trans assay has been used successfully to determine BBB penetrance capabilities of TfR targeting antibodies [31, 32] and was ideally optimised to reliably assess the effect serotransferrin has on the efficacy of antibody transcytosis. Even though the “pulse-chase” portion of the In-Cell BBB-Trans assay was carried out in serum-free media conditions, there was a concern that the bovine transferrin or other molecules present in the three-day differentiation medium (2% FBS) incubation, prior to the assay, may be contributing to a reduced transcytosis output. It has been previously shown that bovine transferrin, which is present in the Foetal Bovine Serum (FBS) used to make the cEND complete and differentiation medium, can bind to mouse TfR and inhibit binding and uptake of endogenous transferrin in mammalian cell lines [53]. To test whether this is the case, cEND cells were incubated with serum-free medium for three days prior conducting the “pulse-chase”, pulsing 13.3 nM RmAb158-scFv8D3 for one-hour, washing and running the chase for six-hours. When comparing the serum-free conditions to differentiation medium (Fig. 3), a small drop in apical recycling was observed. However, and more interestingly, a significant 2.5-fold increase in basolateral transcytosis was observed. These results indicate that a possible inhibitory effect of the FBS on TfR mediated RMT that is initiated already when priming the cells for three days in differentiation medium. Based on these results, all subsequent experiments were carried out with a three-day priming in serum-free medium prior to the “pulse-chase” portion of the In-Cell BBB-Trans assay.

### Improved transcytosis of monovalent, partially-bivalent and bivalent 8D3 antibodies using mouse serotransferrin supplementation

**Fig. 4:**
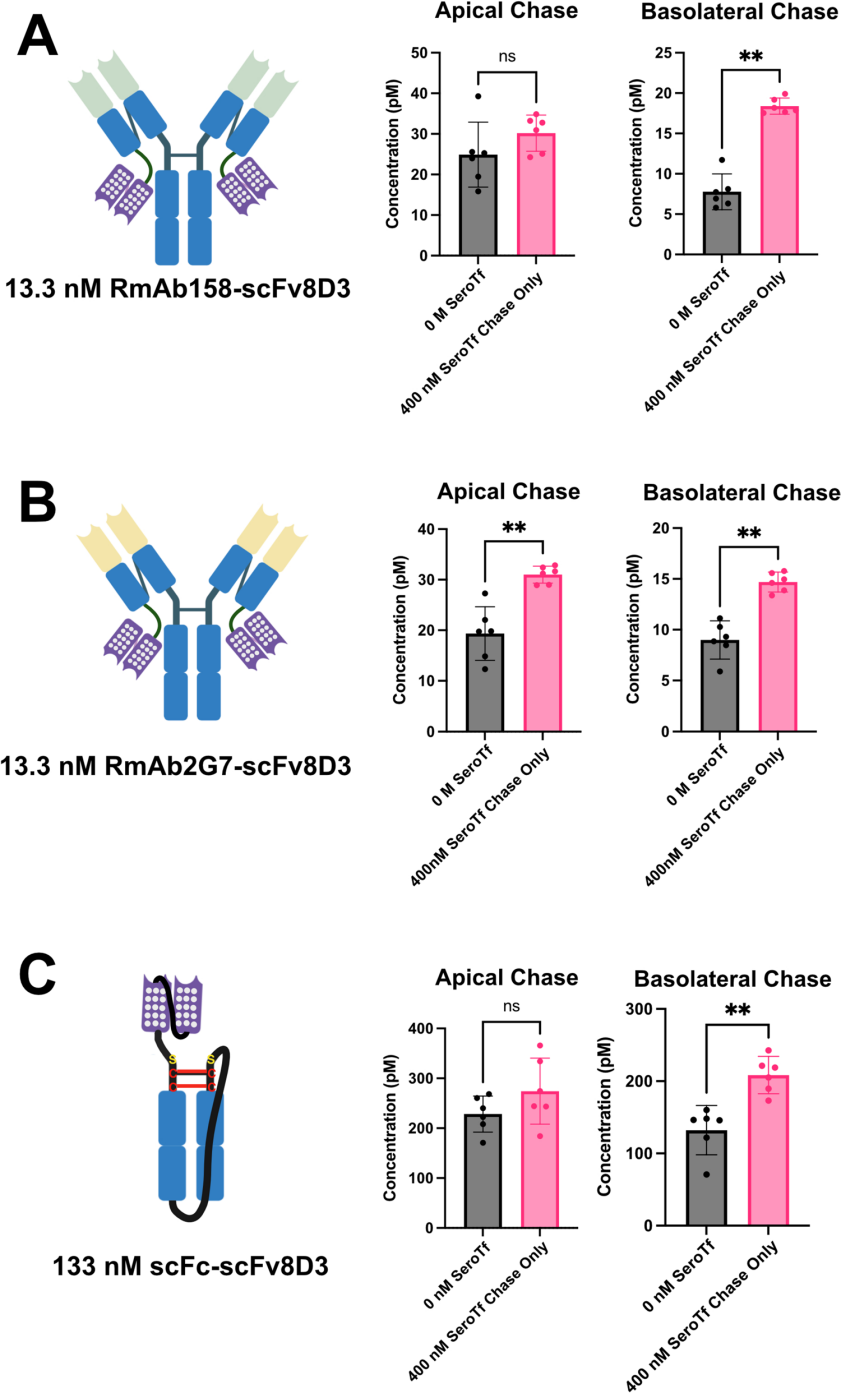
A-C. Graphical representation of average antibody concentrations found in the apical and basolateral 6-h chase compartments of cEND cells plated on 0.4 µm translucent pore Bio-One® 24-well transwell cultures, primed for three-days in serum-free medium and followed by a one-hour “pulse” with 13.3 nM RmAb158-scFv8D3 (Passage 11), 13.3 nM RmAb2G7-scFv8D3 (Passage 11) and 133 nM scFc-scFv8D3 (Passage 28), with or without supplementation of mouse serotransferrin in the chase portion of the assay. Six transwells were used for each pulsed antibody condition (n = 6 technical replicates). The error bars represent 95% confidence intervals. Non-parametric Mann–Whitney pairwise comparisons were conducted as indicated in A-C. ** Represents a significance level of P < 0.01. The polka-dotted regions on the cartoon represent the recombinantly added scFv8D3 that binds to the TfR

To test whether antibodies that utilise TfR-mediated RMT have an improved ability to undergo transcytosis when supplemented with 400 nM mouse serotransferrin, we performed the In-Cell BBB-Trans assay with two partially monovalent/bivalent 8D3 recombinantly added antibodies (RmAb158-scFv8D3 and RmAb2G7-scFv8D3) and one monovalent 8D3 recombinantly added antibody (scFc-scFv8D3) [31, 32], with and without supplementation of mouse serotransferrin to the chase portion of the assay. Both 13.3 nM RmAb158-scFv8D3 and 13.3 nM RmAb2G7-scFv8D3 demonstrated a significant increase in basolateral transcytosis (≈ twofold for both antibodies) when supplemented with 400 nM mouse serotransferrin (Molar Ratio 30:1) (Fig. 4A and B). A moderate increase in apical recycling was also observed, with only RmAb2G7-scFv8D3 demonstrating a significant increase. For the monovalent scFc-scFv8D3, an increased concentration of 133 nM was used, as this has been shown previously to display transcytosis levels similar to that seen with 13.3 nM partially monovalent/bivalent antibodies [31, 32]. Using the same concentration of 400 nM mouse serotransferrin in the chase portion of the assay, and even with a reduced molar ratio of 3:1 when compared to the partially bivalent antibodies, a similar pattern was observed for the monovalent scFc-scFv8D3 antibody supplemented with mouse serotransferrin, with a moderate increase in apical recycling and an almost two-fold significant increase in basolateral transcytosis (Fig. 4C).

### Mouse serotransferrin improves transcytosis of bivalent binding TfR antibodies with mouse or human Fc regions

**Fig. 5:**
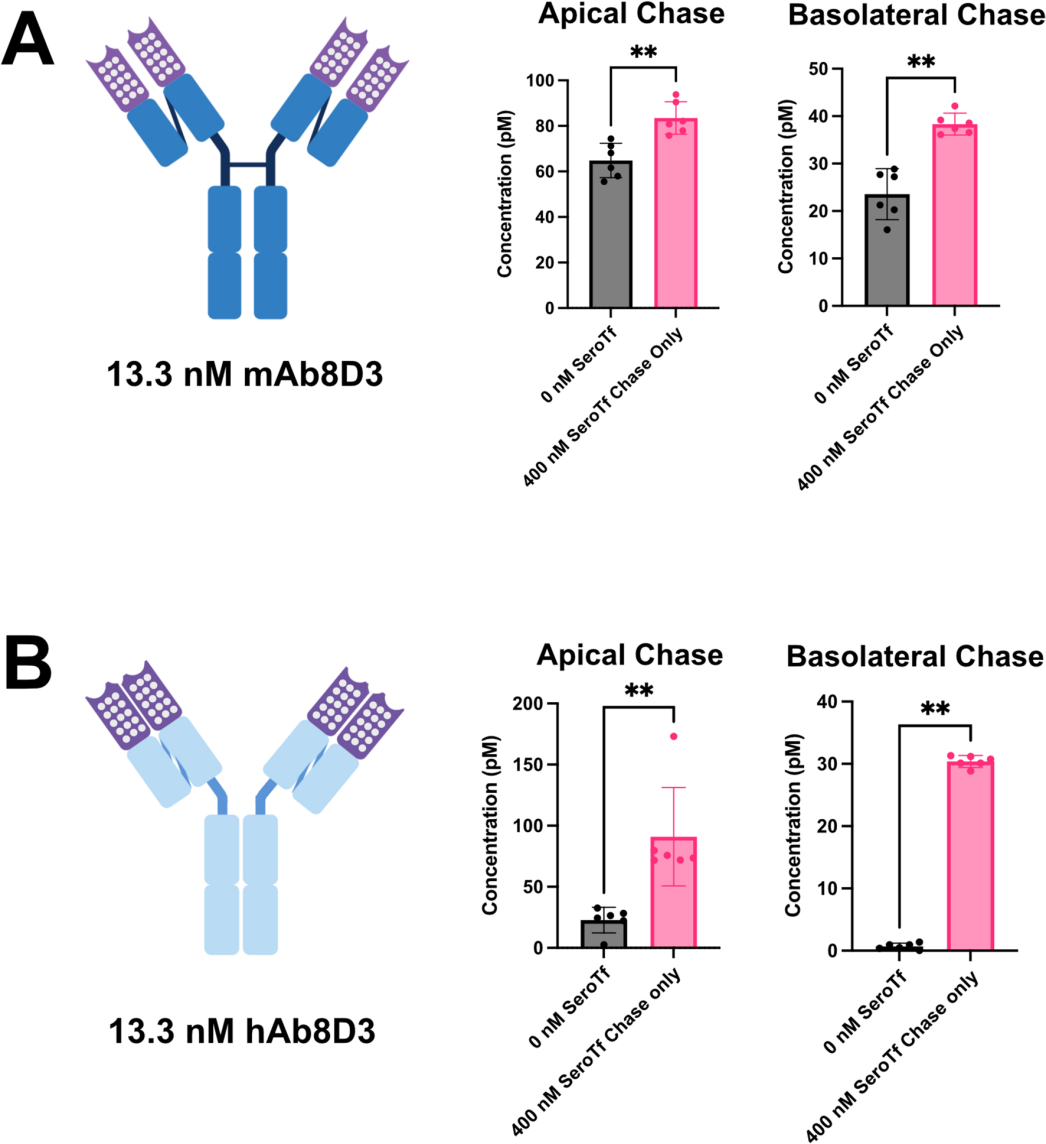
A-B. Graphical representation of average antibody concentrations found in the apical and basolateral 6-h chase compartments of cEND cells plated on 0.4 µm translucent pore Bio-One® 24-well transwell cultures, primed for three-days in serum-free medium and followed by a one-hour “pulse” with 13.3 nM Rat 8D3 TfRmAb (Passage 28) and 13.3 nM Rat 8D3 TfRhAb (Passage 24), with or without supplementation of mouse serotransferrin in the chase portion of the assay. The polka-dotted regions on the cartoon represent the scFv8D3 CDR that binds to the TfR. Six transwells were used for each pulsed antibody condition (n = 6 technical replicates). The error bars represent 95% confidence intervals. Non-parametric Mann–Whitney pairwise comparisons were conducted as indicated in A and B. ** Represents a significance level of P < 0.01

To ensure the efficacy of supplementing TfR-mediated RMT antibodies with mouse serotransferrin was not limited to partially monovalent/bivalent or monovalent designed antibodies, the experiment was repeated with chimeric 8D3 antibodies that either had a mouse Fc (mAb8D3) or a human Fc (hAb8D3). Both bivalent antibodies demonstrated a similar pattern to that seen already with the partially monovalent/bivalent and monovalent antibodies, with a significant increase in both apical recycling and basolateral transcytosis (Fig. 5A and B). Interestingly, the basolateral transcytosis levels of the hAb8D3 with a human Fc was relatively non-existent before the addition of 400 nM mouse serotransferrin to the chase portion of the assay. Taken together, these results highlight a beneficial effect of supplementing TfR-binding antibodies with increased molar concentrations of mouse serotransferrin on in vitro RMT.

### Improved transcytosis of 8D3-independent TfR binding antibodies using mouse serotransferrin supplementation

**Fig. 6:**
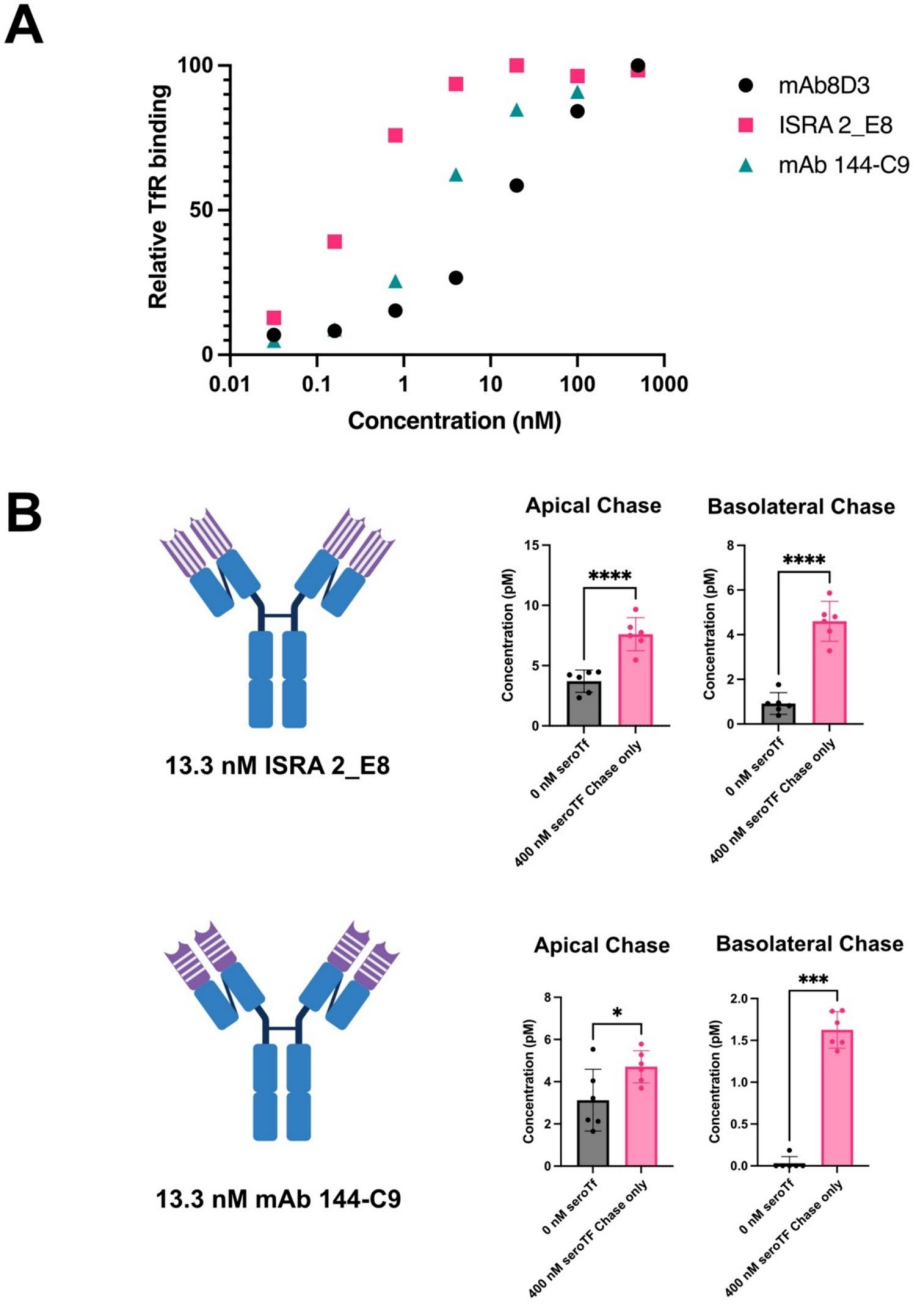
**(A)** ELISA data representing the relative binding efficacy of ISRA 2_E8, mAb 144-C9 and Rat 8D3 mIgG to mouse TfR. NOTE: the 1000 nM data point for mAb 144-C9 is masked by the 1000 nM data points for mAb8D3 and ISRA2_E8. **(B)** Graphical representation of average antibody concentrations found in the apical and basolateral 6-h chase compartments of cEND cells plated on 0.4 µm translucent pore Bio-One® 24-well transwell cultures, primed for three-days in serum-free medium and followed by a one-hour “pulse” with 13.3 nM ISRA 2_E8 (Passage 39) and 13.3 nM mAb 144-C9 (Passage 39), with or without supplementation of mouse serotransferrin in the chase portion of the assay. The vertical (ISRA 2_E8) and horizontal lines (mAb 144-C9) regions on the cartoon represent the CDR that binds to the TfR. Six transwells were used for each pulsed antibody condition (n = 6 technical replicates). The error bars represent 95% confidence intervals. Non-parametric Mann–Whitney pairwise comparisons were conducted as indicated in A and B. * Represents a significance level of P < 0.05. ** Represents a significance level of P < 0.01. *** Represents a significance level of P < 0.001. **** Represents a significance level of P < 0.0001

We have shown that serotransferrin significantly improves transcytosis of TfR-binding-8D3 antibodies. We wanted to see if this enhancement could also be detected using antibodies that bind the TfR in an 8D3-independent manner. Two bivalent mouse IgG TfR-binding antibodies were employed for this study, ISRA 2_E8 and mAb 144-C9. Using a qualitative dose–response ligand-ligand interaction ELISA setup, both antibodies demonstrated a high binding affinity to mouse TfR, compared to mAb8D3 (Fig. 6A). Using the In-Cell BBB-Trans assay, we saw a dramatic decrease in apical recycling and basolateral transcytosis for both ISRA 2_E8 and mAb 144-C9 (Fig. 6B), when quantitatively comparing to the bivalent and partially monovalent/bivalent antibodies previously tested (Figs. 4 and 5). When 400 nM serotransferrin was supplemented during the chase portion of the assay, the apical recycling and basolateral transcytosis was significantly improved. Interestingly, mAb 144-C9, which essentially showed no ability to transcytose, showed a significant improvement in transcytosis following the supplementation of mouse serotransferrin. These results indicate that the addition of mouse serotransferrin greatly enhance apical recycling and basolateral transcytosis of antibodies designed to bind the TfR using receptor-mediated transcytosis mechanisms even though they on their own trancytose poorly.

### *Mouse serotransferrin significantly increases brain uptake of RmAb158-scFv8Da and hAb8D3 *in vivo

**Fig. 7:**
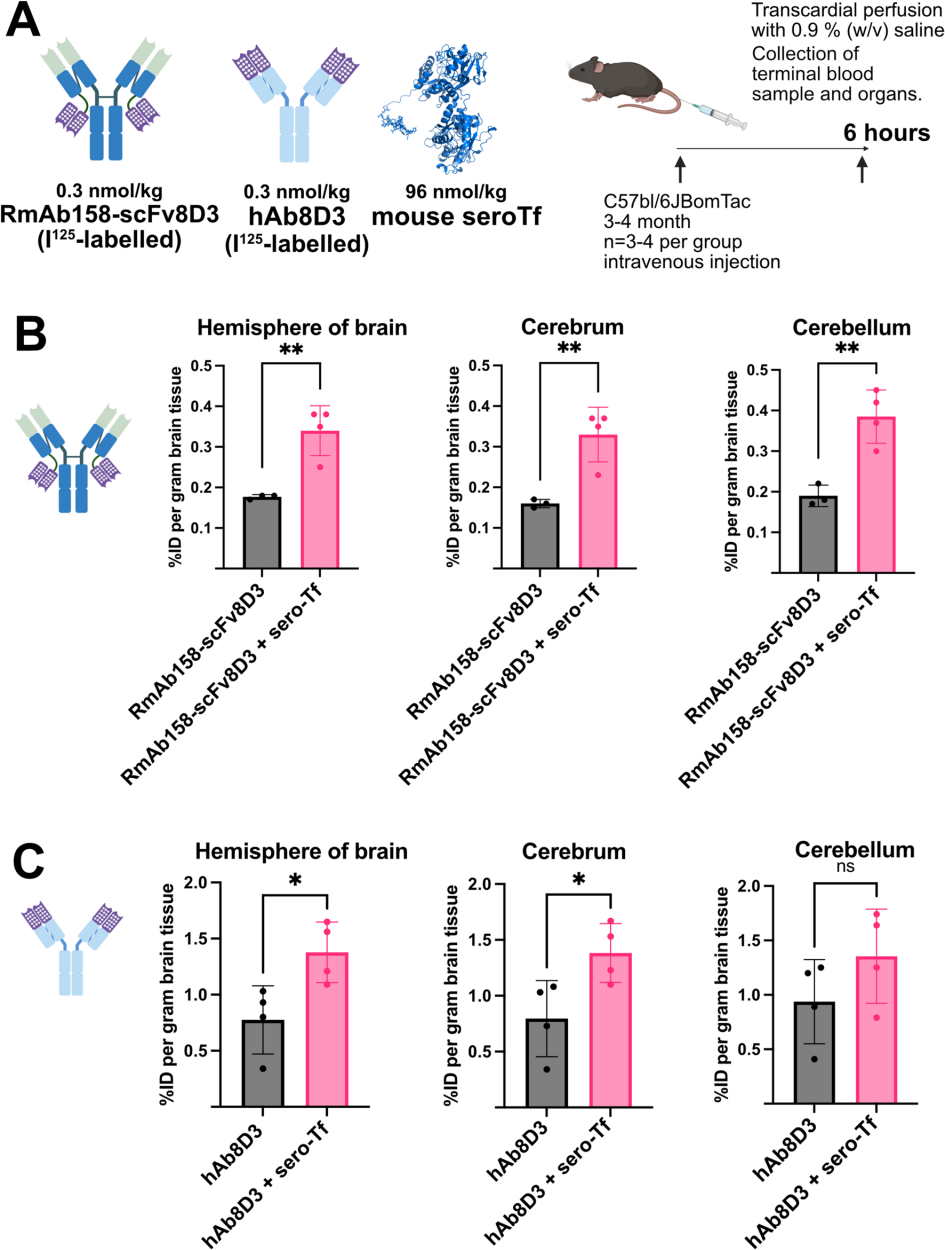
A. Schematic timeline for in vivo experiment to generate data for brain uptake and biodistribution of the constructs. B. In Vivo brain distribution 6-h post-intravenous injection of I^125^ labelled construct at 0.3 nmol/kg RmAb158-scFv8D3 (n = 3) and 0.3 nmol/kg RmAb158-scFv8D3 + 96 nmol/kg mouse serotransferrin (n = 4). C. In Vivo brain distribution 6-h post-intravenous injection of I^125^ labelled construct at 0.3 nmol/kg hAb8D3 (n = 4) and 0.3 nmol/kg hAb8D3 + 96 nmol/kg mouse serotransferrin (n = 4). Uptake in the right hemisphere of the brain, uptake of cerebrum and uptake of cerebellum were analysed. The error bars represent 95% confidence intervals. Significance was taken as P < 0.05 (*) and P < 0.01 (**)

In order to verify the findings of the in vitro In-Cell BBB-Trans assay further, we decided to conduct an in vivo experiment to test whether co-administration of antibody with mouse serotransferrin would improve the efficacy of brain uptake. We used an incubation period of six-hours, to better compare to the 6-h chase used in the in vitro studies. To ensure that the exogenous mouse serotransferrin was large enough to exceed the endogenous serotransferrin levels reported in mammals (25–40 µM) [40], we decided to use a 300-fold mouse serotransferrin to antibody ratio at the time of administration, resulting in 62 µM mouse serotransferrin being delivered to each mouse. This experiment was done with the RmAb158-scFv8D3 and the hAb8D3. The hAb8D3 was not efficient in crossing the in vitro BBB barrier unless mouse serotransferrin was present in the chase-portion of the In-Cell BBB-Trans assay (Fig. 5B). A schematic in vivo experimental setup can be seen in Fig. 7A. The results of the in vivo (Fig. 7B and C) clearly show that co-injection of RmAb158-scFv8D3 with mouse serotransferrin significantly increases uptake in the right hemisphere of the brain, cerebrum and cerebellum by approx. twofold and co-injection of hAb8D3 with mouse serotransferrin significantly increases uptake to 1.8-fold in both the right hemisphere and cerebrum. There is a trend for increased uptake in the cerebellum, but this increase was not found to be significant for hAb8D3. No significant differences were observed in the blood, tissue and organ uptake of recombinant antibodies following the administration of RmAb158-scFv8D3 or hAb8D3, with or without mouse serotransferrin supplementation (Supplementary Fig. 3). These in vivo results show that co-injection of a TfR binding antibody with serotransferrin significantly improves brain uptake.

### Human serotransferrin improves basolateral transcytosis of a monovalent TfR binding antibody

**Fig. 8:**
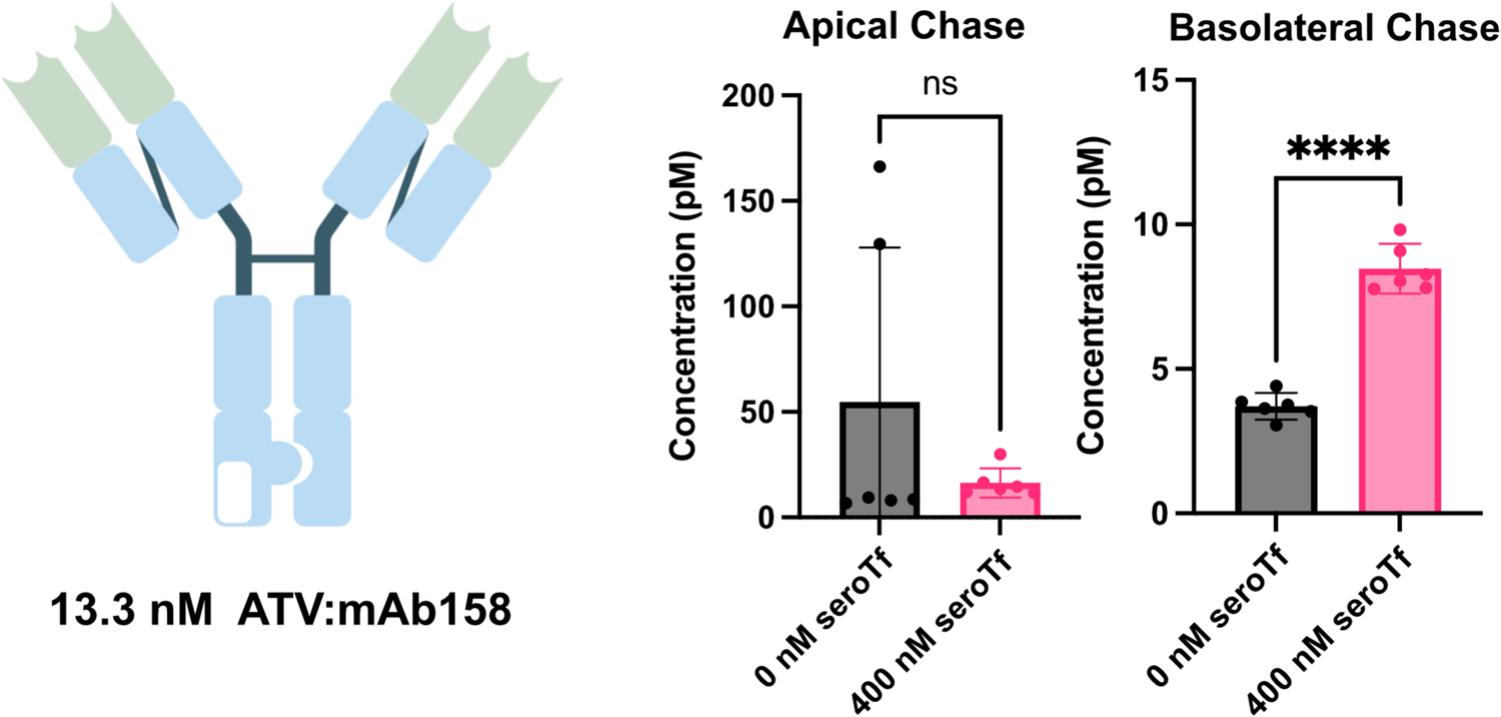
Graphical representation of average antibody concentrations found in the apical and basolateral 6-h chase compartments of hCMEC/D3 cells (Passage 8) plated on 0.4 µm translucent pore Bio-One® 24-well transwell cultures, primed for three-days in serum-free medium and followed by a one-hour “pulse” with 13.3 nM hIgG-15G11-1 and 13.3 nM hIgG 158-bs-denali, with or without supplementation of human serotransferrin in the chase portion of the assay. The white rectangle in the Fc portion of the antibody in the cartoon represents the TfR binding region. Six transwells were used for each pulsed antibody condition (n = 6 technical replicates). The error bars represent 95% confidence intervals. Non-parametric Mann–Whitney pairwise comparisons were conducted as indicated in A and B. **** Represents a significance level of P < 0.0001

To see if the effect of serotransferrin can be transferred to studies in humans, a modified human In-Cell BBB-Trans assay was performed using hCMEC/D3 cells and a human IgG based monovalent TfR binding antibody (ATV:mAb158) known to cross the human BBB. Similarly to the murine in vitro and in vivo studies, the ATV:mAb158 demonstrated a highly significant increase in basolateral transcytosis when supplementing the chase portion of the assay with human serotransferrin, with no observable difference indicated when assessing apical recycling (Fig. 8). Data shown in this manuscript indicates that human serotransferrin does act like the mouse counterpart in improving the transcytosis of TfR binding antibodies in a human in vitro BBB model.

## Discussion

Our group, along with many others, have successfully delivered TfR-binding-8D3 antibodies non-invasively into the brain milieu of both wildtype and transgenic mice via TfR-mediated RMT pathways [6, 12, 14, 19, 20, 33, 35, 42, 43, 45, 50, 54].

Given the endogenous Fe-Tf transport via TfR, we aimed to test whether supplementing with serotransferrin would further increase the transcytosis levels of co-administered TfR-binding antibodies. We designed and produced serotransferrin protein (Fe-Tf) that bound mouse TfR, with a reduced binding affinity observed for the human TfR.

To ensure that the bovine Tf present in the fetal bovine serum (FBS), commonly used in the cell media, did not interfere with our experiments, we removed it. Upon removal, we observed that FBS had a blocking effect on TfR-mediated transcytosis (Fig. 3). This could be due to several factors, possibly including an antagonistic effect of bovine serotransferrin or the resulting Tf starvation of the cells. Previous studies have shown that bovine Tf binds to TfR2 and competes with the binding of human Tf [23]. Bovine Tf has been reported to have no or low binding affinity to TfR1[23], which is the primary receptor used for TfR transcytosis across the blood–brain barrier (BBB). Therefore, it is likely that the effect is indirect, possibly by reducing the amount of TfR1 on the cell surface. Given that FBS contains many components, it is also possible that other molecules present in FBS contribute to the inhibition of TfR transcytosis or that the starvation of certain molecules upon removal causes an increased transcytosis.

Using the standardised murine in vitro BBB model system (In-Cell BBB-Trans assay) capable of quantitatively determining antibody transcytosis [31, 32], we demonstrated that a 30:1 molar ratio of mouse serotransferrin (added in the chase) to antibody (added in the pulse) contributed to an approximate twofold increase in transcytosis of antibodies that bind to the TfR in a partially monovalent/bivalent fashion (RmAb158-scFv8D3 and RmAb2G7-scFv8D3). We also saw a similar improvement in transcytosis when using a 3:1 molar ratio of mouse serotransferrin to an scFv of the 8D3 antibody designed to bind monovalently to the TfR (scFc-scFv8D3), further validating the use of mouse serotransferrin as a transcytosis enhancement supplement.

It is to us not perfectly clear by which mechanism serotransferrin enhances the uptake. It can be that the TfR binder is bound to TfR on the endothelial cell surface and when later the serotransferrin is added it binds to the same TfR and this induces the endocytosis of this complex. Since the serotransferrin is added in the chase after the excess of the antibodies has been washed away it is unlikely that it is a change in the concentration of the TfR on the cell surface that is the cause. When the Tf- TfR complex is endocytosed it can be sorted through different pathways one being to the lysosome and degraded and another to transcytosis[48]. The addition of Tf in the chase might affect this intracellular sorting so that more is transcytosed.

In addition to the recombinant scFv8D3 antibodies, we were able to show a similar increase in transcytosis levels of purely bivalent TfR binding mouse and human antibodies (mAb8D3 and hAb8D3) when using a 30:1 molar ratio of serotransferrin to antibody. Interestingly, the addition of a 30:1 molar ratio of mouse serotransferrin to hAb8D3 significantly improved the miniscule levels of transcytosis observed when administering the antibody alone by more than 40-fold. One hypothesis that could account for the difference between the human and mouse antibodies is the presence of the mouse neonatal Fc receptor (FcRN) on the cEND cell membrane. It is possible that during endocytosis of the antibody-TfR complex, the Fc region of the antibody also binds FcRN that is endocytosed along with the antibody-TfR complex. It has been shown previously that IgG antibodies bound to the FcRN escape lysosomal degradation pathways and instead IgG antibodies are recycled or undergo transcytosis [26, 39, 44, 52]. Being that FcRn expression has been previously reported on brain microvascular endothelium [46], it is not unreasonable to hypothesize that a FcRn-Antibody-TfR complex forms in the early endosome following endocytosis, with the FcRn performing a protective function whereby the antibody escapes lysosomal degradation pathways and instead undergoes transcytosis. In the situation where the hAb8D3 is used, the Fc portion of the antibody cannot bind or has a reduced binding affinity to the mouse FcRN receptor, resulting in a lack of protection from lysosomal degradation, subsequently leading to minute levels of transcytosis. Upon addition of mouse serotransferrin, the benefits of binding the FcRN is minimized and alternate pathways drive transcytosis instead of lysosomal degradation, leading to elevated transcytosis levels. Using mouse antibodies, the combination of binding to the FcRn receptor and the amplification properties of mouse serotransferrin supplementation leads to an even larger proportion of antibodies undergoing transcytosis. Using human antibodies, mouse serotransferrin supplementation overrides the FcRN response, leading to the transcytosis of the human antibody. Further studies on the ability of human IgG Fc regions to bind the mouse FcRn on BBB endothelial cells needs to be determined, both in vitro and in vivo, in order to start corroborating the aforementioned hypothesis.

To corroborate the findings conducted in vitro showing improved BBB transcytosis of antibodies supplemented with mouse serotransferrin, i*n vivo* experiments were performed using the TfR-binding-8D3 human antibody (hAb8D3), as the ability of this antibody to undergo transcytosis using the In-Cell BBB-Trans assay was more or less non-existent in the absence of mouse serotransferrin (Fig. 6B). In addition, we needed a strategy to administer a concentration of mouse serotransferrin that would not be drowned out by endogenous plasma levels of serotransferrin that is known to be high. We decided to further increase the molar ratio of administered mouse serotransferrin to antibody to 300-fold, in order to further increase serotransferrin levels within the mice, thereby improving the chances of seeing an enhanced brain uptake effect when co-administering the antibody with mouse serotransferrin. The concentration of administered mouse serotransferrin (approximately 62 µM) at time of injection, exceeded the average concentration of serum transferrin reported in mammals [40], which is estimated to range between 2.5–3.6 mg/ml (approximately 25–40 µM). This strategy was successful and we were able to detect a 1.8-fold increase inuptake in the right hemisphere of the brain when comparing co-administered antibody and mouse serotransferrin to antibody administered alone. The fact that antibody alone did cross the BBB in vivo, but did not show an ability to cross the endothelial layer in vitro without mouse serotransferrin supplementation, could be explained by the presence of endogenous serotransferrin within the mice that led to the unexpected uptake into the brain. Regardless, increasing the endogenous levels of mouse serotransferrin did result in a significantly improved uptake of the antibody into the brain milieu and it would be interesting to repeat these experiments in an in vivo murine system that is devoid, or has extremely reduced levels, of serotransferrin. One possible explanation for why we did see an effect in vivo despite the already high in vivo levels of serotransferrin could be that the endogenous and recombinant serotransferrins have different posttranslational modifications, which possibly can affect the function of the serotransferrin. It has been reported that serotransferrin is commonly glycosylated, which affects its binding to iron[17, 28, 29], but other posttranslational modifications could also have effects.

Further we also tested supplementing RmAb158-scFv8D3 with serotransferrin in vivo using the same experimental set up. The levels of transcytosis were doubled when supplementing with serotransferrin further confirming that a slight increase to the endogenous levels of transferrin still causes a significant enhancement of the uptake to the brain.

In summary, the need for developing therapeutics that can non-invasively penetrate the BBB, bind to a target in the brain parenchyma and trigger an efficacious response, is of vital important considering neurodegenerative diseases, such as Alzheimer’s disease, frontotemporal dementia and synucleinopathies, are some of the leading causes for mortality and morbidity around the World [11]. Being that a large proportion of neurodegenerative disorders are identified by disease-specific protein accumulation [8], biologics are being employed to target proteins that accumulate and ameliorate the associated pathology. Advances are being made in developing efficacious biopharmaceuticals, but challenges remain relating to non-invasively delivering large macromolecules into the brain via the BBB. We have discovered that enhancing TfR-mediated murine and human RMT through serotransferrin supplementation improves the efficacy of TfR-binding antibody transcytosis across the BBB and subsequent brain uptake. This supplement could create improved treatment regimens that target pathological proteins within the brain parenchyma.

## Methods

### Design of mouse serotransferrin

Mouse serotransferrin (Ensembl—ENSMUST00000112645.8) was cloned into the vector pcDNA3.4 (Gene Art) with a signal peptide (MSVPTQVLGLLLLWLTDARC) as well as a 6xHis-tag (HHHHHH) and a myc-tag (EQKLISEEDL) at the N-terminal. A short linker (PGGGSP) was inserted between the myc-tag and the serotransferrin sequence (Supplementary Fig. 1).

### Expression and purification of the mouse and human serotransferrin

The mouse serotransferrin (Mouse Sero-Tf (his-myc)) used in the experiments was expressed and purified according to earlier published work [13, 20] using Expi293 cells (Thermofisher cat. no. A14527) transiently transfected with pcDNA3.4 vectors using polyethylenimine (PEI – Polyscience cat. no. 24765–1) as the transfection reagent. The protein was purified on a Nickel column (Cytiva cat. no. 17371206) and eluted with 0.5 M Imidazole (Millipore cat. no. 1.04716.0250). The buffer was exchanged to PBS (Thermofisher cat. no. 14190250) immediately after elution and the protein concentration was determined at A280. Human Holo-transferrin (Human Holo-Tf) was purchased (Sigma T0665) and dissolved to a concentration of 1 mg/ml in PBS, before being filtered through a 0.22 µm sterile syringe filter.

### Confirmation of purity and size of the recombinant monoclonal antibodies and mouse serotransferrin

The antibodies were mixed with LDS sample buffer (Life Technologies cat. no. B0007) and loaded onto 4–12% Bis–Tris protein gels (Invitrogen cat. no. NW04125BOX). The gel was then stained with PAGE blue protein solution (Thermo Scientific cat. no. 24620) using PageRuler™ Plus Prestained Protein Ladder, 10 to 190 kDa (Thermo Scientific cat. no. 26619) as a molecular weight standard. Images of the stained gel were taken using an Odyssey Fc Machine (LI-COR Biosciences).

### Description of the mouse- and human-based antibodies

Eight antibodies were used throughout this study, and unless otherwise stated, purified according to earlier published work [13, 20] using Expi293 cells (Thermofisher cat. no. A14527) transiently transfected with pcDNA3.4 vectors using polyethylenimine (PEI – Polyscience cat. no. 24765–1) as the transfection reagent. 1. The RmAb158-scFv8D3 with a murine IgG2C constant part [20]—(Mw 203 kDa) selectively binds to Ab protofibrils via the CDR of the variable heavy and light chains of the RmAb158 antibody [9]. The scFv of the 8D3 antibody, which selectively binds to TfR [3], was attached using an 11 amino acid linker to the C-terminus of the RmAb158 light chain [20]. 2. The RmAb2G7-scFv8D3 with murine IgG2C Fc part- (Mw 200 kDa) selectively binds to High mobility group box 1 proteins (HGMB1) via the CDR of the variable heavy and light chains of the RmAb2G7 antibody [27]. The scFv8D3 protein, was attached as above to the C-terminus of the RmAb2G7 light chain (Morrison, Petrovic, et al., 2023). 3. The scFc-scFv8D3 antibody (Mw 82 kDa) is a single chain of the CH1 and CH2 domains of the IgG2c Fc part and has the same binders as the unmodified Fc. The scFv8D3 sequence was connected to the N-terminus of the scFc region of a murine IgG2c antibody using an 11 amino acid linker [31, 32]. 4. The 8D3(from rat) with a murine IgG2c constant part (mAb8D3 – Mw 146 kDa) selectively binds to murine TfR and has a murine IgG2 [4]. The variable region of the heavy chain and light chain of 8D3 were fused to the constant region of mouse IgG2c and mouse kappa light chain respectively. 5. The human-rat chimeric 8D3 (hAb8D3 – Mw 145 kDa) has the same rat 8D3 that binds the murine TfR [4], but on a human Fc part. The variable region of the heavy chain and light chain of the 8D3 were fused to the constant region of human IgG1 and human kappa light chain respectively. 6. The mAb144-C9 monoclonal murine IgG-based antibody (Mw 148 kDa) selectively binds to TfR via the CDR of the variable heavy and light chain of the antibody. The scFv of mAb 144-C9 was originally developed by Yumab GmbH (Braunschweig, Germany) by phage display to bind the murine TfR (murine peptide sequence QDVKHPVDGKSLYRDSN). 7. The ISRA 2_E8 monoclonal monoclonal murine IgG-based antibody (Mw 146 kDa) selectively binds to TfR via the CDR of the variable heavy and light chain of the antibody. The scFv of ISRA 2_E8 was originally developed by BioArctic AB to bind the murine TfR. 8. The ATV:mAb158 human IgG-based antibody (Mw 147 kDa) selectively binds to Ab protofibrils via the CDR of the variable heavy and light chains of the antibody of the antibody [22]. The antibody is engineered to have a monovalent TfR binding site incorporated into the Fc domain of the human IgG-based antibody. Confirmation of antibody molecular weight and purity is shown in the SDS-PAGE analysis represented in Supplementary Fig. 2.

### *Assessing *in vitro* binding to mouse and human TfRs*

Binding of the purified mouse serotransferrin, was assessed using a modified, previously published, ELISA method [47]. Briefly, 96-well half area plates (Corning Incorporated cat. no. 3960) were each coated with serial dilutions of the recombinant mouse serotransferrin in PBS overnight at 4 °C. After blocking for 2-h at room temperature with 1% BSA (Sigma cat. no. A7030) in PBS, 50 ng of recombinant mouse or human TfR protein were added and incubated for 2-h at RT while shaking. For the detection, a one-hour incubation at RT with StrepMAB-Classic (IBA Lifesciences GmbH 2–1507-00) was used, followed by a one-hour incubation at RT with horse-radish peroxidase (HRP) conjugated secondary goat anti-mouse antibody (Sigma cat. no.12349). Signal development was performed with K-blue aqueous TMB (Neogen Corp cat. no. 331177). The absorbance was measured at 450 nm using a Spark® multimode microplate reader (Tecan). All dilution series (except the coated protein) were made in ELISA incubation buffer (1 × PBS with 0.1% BSA and 0.05% Tween-20 (Sigma cat. no. P9416)) and the wells were washed between each step with ELISA washing buffer (1 × PBS with 0.05% Tween-20). Binding of the purified ISRA 2_E8, mAb144-C9 and mAb8D3 to mouse TfR was assessed using an almost identical ELISA protocol, but for two changes. The first change was that the 96-well half area plates were coated overnight at 4 °C with 50 ng mouse TfR, followed by a 2-h room temperature blocking step. The second was that serial dilutions of each antibody were added to each well and incubated for 2-h at room temperature while shaking. Detection, development and absorbance measurement was carried out as stated above. Relative binding affinity to mouse or human TfR was performed using the Normalize function in Prism 9 for macOS (9.3.1).

### *Determination of *in vitro* BBB transcytosis of the recombinant monoclonal antibodies with and without serotransferrin supplements*

The previously described In-Cell BBB-Trans assay (Morrison, Petrovic, et al., 2023), along with a modified protocol using human endothelial cells, were used to determine the transcytosis efficiency of murine TfR binding antibodies (RmAb158-scFv8D3, RmAb2G7-scFv8D3 scFc-scFv8D3, mAb8D3, hAb8D3, ISRA 2_E8 and mAb 144-C9) and human TfR binding antibody (ATV:mAb158) respectively, in the presence of mouse or human serotransferrin. In short, ninety thousand murine cerebral endothelial cells (cEND—Applied Biological Materials T0290) or Human Brain Microvascular Endothelial Cells (hCMEC/D3) were plated onto Greiner Bio-One Thincert™ translucent (1 × 108 pores/cm2) PET membranes (Transwell) with high density 0.4 µm pores in 24-well cell culture plates (BioNordika 662,640) and incubated for four hours in complete cEND medium (DMEM (cat. no. 11960044) supplemented with 10% FBS (cat. no. 10270106), 1X non-essential amino acids (cat. no. 11140–050), 1X Glutamax (cat. no. 35050061), 1 mM sodium pyruvate (cat. no. 11360039) and 10 U/ml Penicillin/Streptomycin (cat. no. 15140122)—all media and supplements were from Gibco™) or EGM™ −2 MV Microvascular Endothelial Cell Growth Medium-2 supplemented with 5% FBS (BulletKit™ Lonza CC-3202) respectively at 37 °C and 5% CO2. The plated mouse and human cells were re-fed with serum-free medium (same medium as previously described, but with the FBS removed) and left for an additional 72-h at 37 °C and 5% CO2. The transwells were pulse-incubated apically with of RmAb158-scFv8D3 (13.3 nM), RmAb2G7-scFv8D3 (13.3 nM) scFc-scFv8D3 (133 nM), mAb8D3 (13.3 nM), hmAb8D3 (13.3 nM), ISRA E2_8 (13.3 nM), 144 C9 (13.3 nM) and ATV:mAb158 (13.3 nM) in serum-free conditions at 37 °C and 5% CO2 for one hour. Volumes used for the pulse apical and basolateral chambers, 75 µl and 400 µl respectively, were collected to corroborate the starting concentration of the antibodies used and determine the barrier properties of the endothelial cells (Pulse samples). The monolayers were washed at room temperature in serum-free medium apically (400 µl) and basolaterally (800 µl) three times, with the final wash collected to monitor efficiency of removal of the unbound antibodies (Wash samples). Serum-free medium with and without species specific serotransferrin was added to the apical (100 µl) and basolateral (400 µl) chambers. The cultures were incubated at 37 °C and 5% CO2 for six hours, upon which time, entire apical and basolateral volumes were collected to assess the recycling and transcytosis of the antibodies into the apical and basolateral chambers respectively (Chase samples). Unless otherwise stated, six transwells were used for each pulsed antibody condition (n = 6 technical replicates).The cEND and hCMEC/D3 cells used in all described experiments were between passages 11–28 and 8–11 respectively. The cells were monitored weekly for viability and cell growth. In addition, bi-annual myoplasma testing on the cell supernatant was performed to ensure the absence of bacterial contamination in the stock endothelial cells used to set up the In-Cell BBB-Trans assay. No further authentication was performed in the laboratory other than those previously mentioned.

### Analysis of media samples from the In-Cell BBB-Trans assay

Analysis of the Pulse, Wash and Chase samples of the In-Cell BBB-Trans assay was performed using a previously described ELISA (Morrison, Petrovic, et al., 2023). In short, 96-well full-well ELISA plates (Sarstedt) were coated with PBS diluted 1/5000 Goat-anti Mouse IgG, F(ab’)2 fragment specific antibody (JacksonImmunoResearch cat. no.115–005–006—RmAb158-scFv8D3, RmAb2G7-scFv8D3, mAb8D3, ISRA E2-8 and 144 C9 samples), 1/5000 Goat-anti Mouse IgG, Fcγ fragment specific (JacksonImmunoResearch Cat. No. 115–005–008—scFc-scFv8D3 samples) or 1/5000 AffiniPure Goat-anti-Human IgG F(ab')2 fragment specific (Jackson Immunoresearch 109–005–097 – hAb8D3 and ATV:mAb158 samples) and incubated at 4 °C overnight. Diluted and undiluted apical and basolateral samples from the In-Cell BBB-Trans assay, along with known standard concentrations of monoclonal antibodies, were added to the wells and incubated for two-hours at room temperature on a 500-rpm shaking platform. For detection of the mouse antibodies, a horse-radish peroxidase (HRP) conjugated secondary goat anti-mouse antibody (Sigma cat. no.12349) was used. For the human antibody, a HRP conjugated secondary goat anti-human antibody Goat (1/10,000—Jackson Immunoresearch 109–035–088) diluted in ELISA incubation buffer was used. Following a one-hour room temperature incubation with the detection antibodies, the signal was developed with K-blue aqueous TMB (Neogen Corp cat. no.331177). The absorbance was measured at 450 nm using a Spark® multimode microplate reader (Tecan). All dilution series (except the coated protein) were made in ELISA incubation buffer (1X PBS (Thermofisher cat. no. 18912014 with 0.1% BSA (Sigma cat. no. A7030) and 0.05% Tween-20 (Sigma cat. no. P9416)) and the wells were washed between each step with ELISA washing buffer (1 × PBS with 0.05% Tween-20). The wells were washed between each step with ELISA washing buffer (1X PBS with 0.05% Tween-20). Statistical analysis between indicated populations was performed using the 1-way ANOVA and Mann–Whitney statistical analysis function in Prism 9 for macOS (9.3.1). The minimal accepted significance level was P ≤ 0.05 (see Statistical analysis section for detailed description of analyses performed).

### Radiochemistry

Rmab158-scFv8D3 and hAb8D3 were labelled with Iodine-125 (125I, Perkin Elmer Inc, UK) for in vivo analysis as described previously [18, 42, 43]. The antibodies were mixed with 125I and labelling was performed by using direct ionization of 125I with 1 mg/mL Chloramine-T (Sigma cat. no. 857319) in PBS (Thermofisher cat. no. 14190250). The reaction was stopped after 90 s by adding 1 mg/mL Sodium meta-bisulphite (Sigma cat. no. 08982). Radio-labelled recombinant proteins were purified from free and unbound iodine by using Zeba mini desalting columns (Thermofisher cat. no. 89883), followed by elution with PBS for buffer exchange. The radio-labelling was always performed a maximum 2 h before the experiment. The labelling yield was between 60–70% and was calculated based on the amount of 125I that was initially added and on the remaining activity of the labelled protein after buffer exchange. 125I labelled recombinant proteins were administered with a dose of 0.3 nmol/kg.

### Animals

In this study, C57Bl/6JBomTac mice (males) were used and purchased via a certified supplier (Taconic M&B). Mice were housed in an animal facility at Uppsala University. The animals had free access to water, food and housing material under controlled temperature and humidity. The male’s weight was between 26.8 – 31.2 g. The animals were stalled with 3–4 animals per cage in individually ventilated cages. All procedures were carried out according to the Swedish ethical policies regarding animal experiments. The ethical permit was approved by the Uppsala County Animal Ethics Board (# 5.8.18–04903-2022). Animals were under closely surveillance during the described in vivo experiment and at no point did the mice exhibit any signs of ill health or adverse side-effects to the administered radiolabelled antibodies and proteins.

### Brain uptake studies and biodistribution in wild-type mice

C57Bl6JBomTac wild-type mice (3–4 months of age, n = 3–4) were intravenously injected into the tail vein with 0.3 nmol/kg RmAb158-scFv8D3 or 0.3 nmol/kg RmAb158-scFv8D3 plus 96 nmol/kg mouse serotransferrin or 0.3 nmol/kg hAb8D3 or 0.3 nmol/kg hAb8D3 plus 96 nmol/kg mouse serotransferrin. The mice were euthanized by transcardial perfusion with 0.9% (w/v) NaCl (Merck cat. no. 1.06404.1000) under deep anaesthesia with isoflurane six-hours (0.3 nmol/kg hAb8D3 and 0.3 nmol/kg hAb8D3 plus 96 nmol/kg mouse serotransferrin) post injection. No randomization or blinding was used, but different experimental groups were distributed equally among the cages. No sample size calculation was used. Terminal blood was collected from the heart prior to transcardial perfusion and plasma was separated from the blood cells by centrifugation at 15.000 × g for 5 min. Perfused brains, peripheral organs (liver, spleen, heart, lung, kidney, pancreas, thyroid) and tissues (muscle, bone, skull) were isolated. The right hemisphere was kept intact, whereas the left hemisphere was dissected in cerebrum and cerebellum.The radioactivity levels in dissected brain regions, peripheral organs, tissues and blood samples were measured using a gamma counter (WIZARD 1480, Wallac Oy, Turku, Finland) as previously described [42, 43]. Based on this measured radioactivity (counts per minute, CPM), the concentration of the radioactivity was quantified as percentage of the injected dose (%ID) per gram tissue or blood. The injected dose was calculated as follows: Injected dose = Activity in Syringe before injection [MBq]*EXP(-LN(2)/85536 [min]*Time difference to injection time before [min])-Activity in syringe after injection [MBq]/(EXP(-LN(2)/85536 [min]*Time difference to injection times after [min])) and injected activity per animal weight = MBq/g body weight. Statistical analysis between indicated populations was performed using the Mann–Whitney statistical analysis function in Prism 9 for macOS (9.3.1) and the minimal accepted significance level was P ≤ 0.05 (see Statistical analysis section for detailed description of analyses performed).

### Statistical analysis

Two-tailed post-hoc power analysis using G*Power Version 3.1.9.6. [10] was performed to evaluate power values for both sets of experiments conducted in vitro and in vivo. Effect size (d) was calculated using pairwise mean and standard deviation comparisons that demonstrated a statistical significance. The α error probability was set to 0.05 and the sample size number stated was used to determine the final power value (1-β error probability). Unless otherwise indicated, the power values obtained for all experiments conducted in vitro and in vivo obtained at least 80% (1-β > 0.8). Not all determined values demonstrated a normal Gaussian distribution using an α value of 0.05. For this reason, we preferred to use non-parametric analyses for all data points. A non-parametric two-tailed Mann–Whitney test was used to determine statistical differences for values obtained using the In-Cell BBB-Trans assay in Figs. 3, 4, 5, 6 and 8. A non-parametric two-tailed Mann–Whitney was used to determine statistical differences between the values obtained between the antibodies delivered with and without mouse serotransferrin in the in vivo brain uptake studies (Figs. 7). No test for outliers was performed for any of the comparisons.

## Supplementary Information

Below is the link to the electronic supplementary material.

Supplementary file1 (JPG 1868 KB)

Supplementary file2 (JPG 836 KB)

Supplementary file3 (JPG 330 KB)

## Data Availability

Data is provided within the manuscript or supplementary information files. Possible additional data are available to the corresponding author upon reasonable request.
