# Supplementary figures and images for: Serotransferrin enhances transferrin receptor-mediated brain uptake of antibodies
Source: Drug Deliv Transl Res. 2025 Feb 19;15(9):3321–37. doi: 10.1007/s13346-025-01811-1 (PMC12350589; doi:10.1007/s13346-025-01811-1)

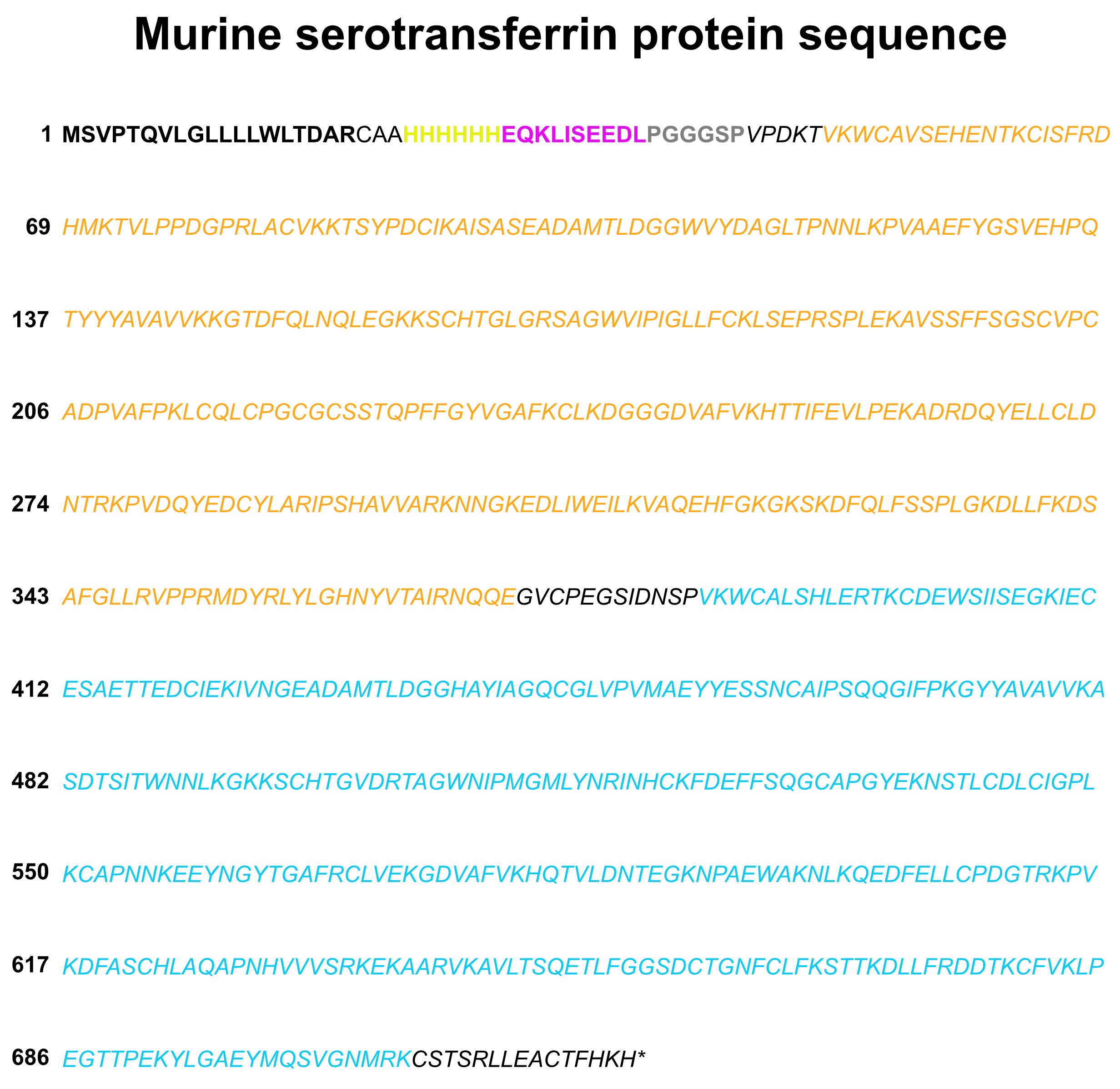

Supplement: Supplementary file 1 — Supplementary file1 (JPG 1868 KB) [file 13346_2025_1811_MOESM1_ESM.jpg]

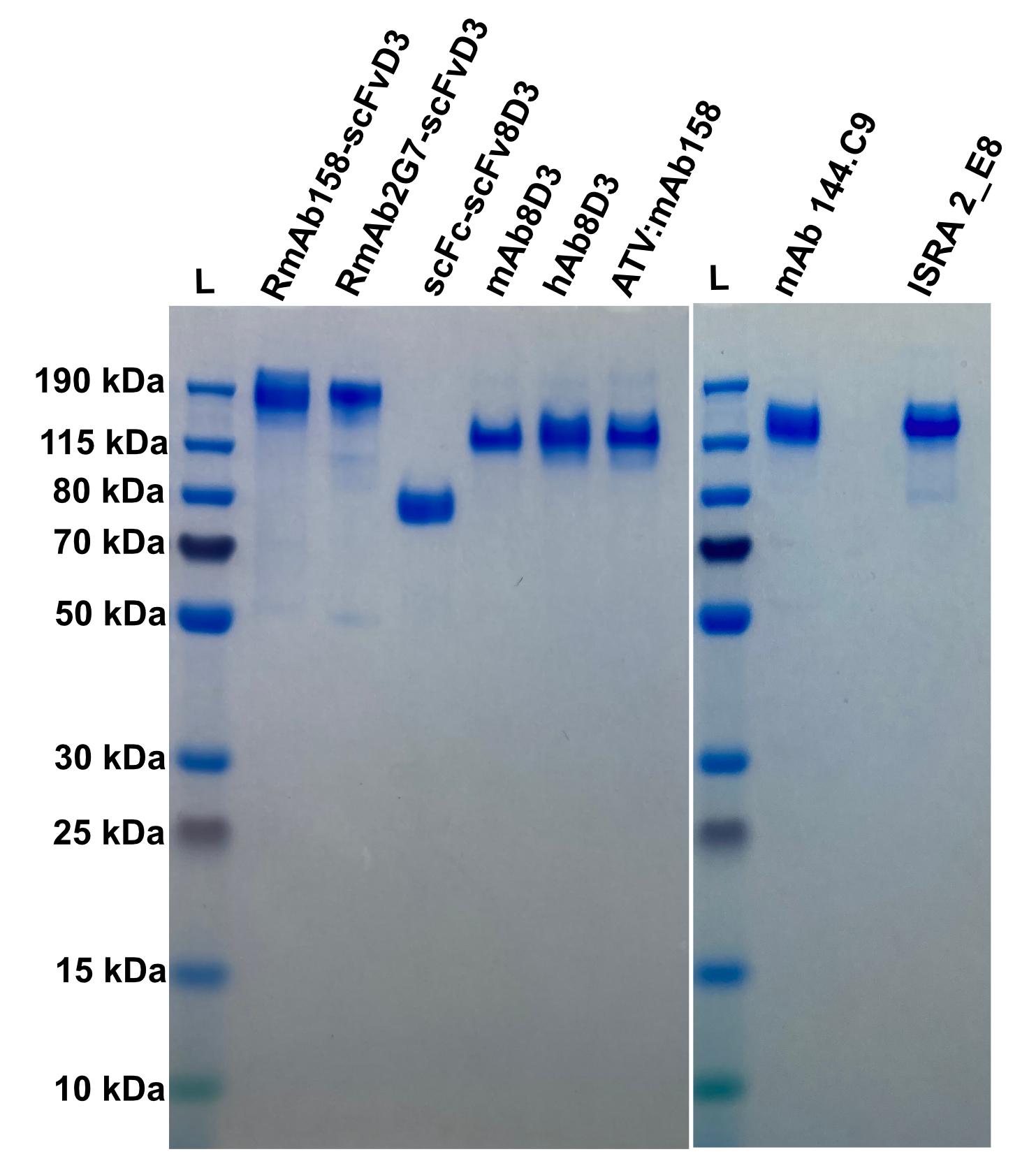

Supplement: Supplementary file 2 — Supplementary file2 (JPG 836 KB) [file 13346_2025_1811_MOESM2_ESM.jpg]

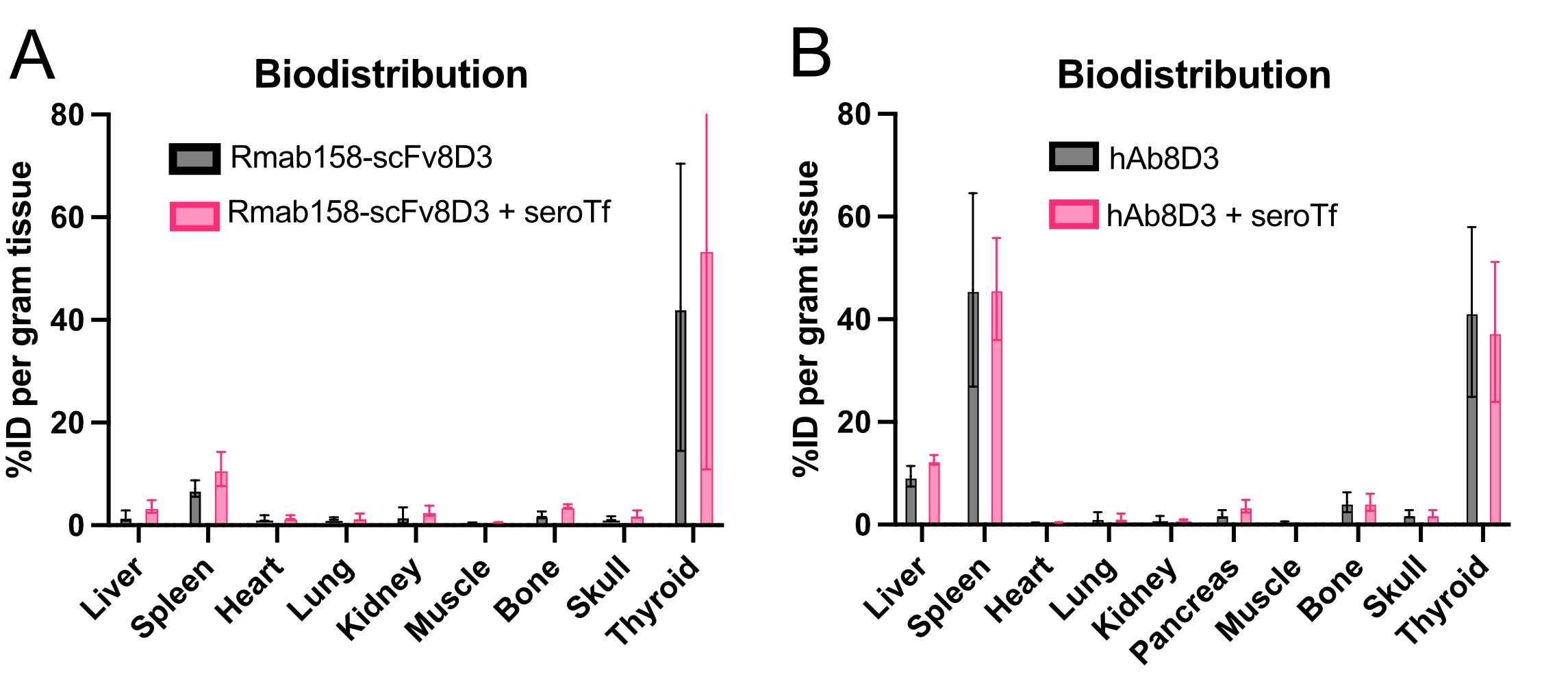

Supplement: Supplementary file 3 — Supplementary file3 (JPG 330 KB) [file 13346_2025_1811_MOESM3_ESM.jpg]
